# IL-4/IL-13 independent goblet cell hyperplasia in experimental helminth infections

**DOI:** 10.1186/1471-2172-9-11

**Published:** 2008-03-28

**Authors:** Reece G Marillier, Chesney Michels, Elizabeth M Smith, Lizette CE Fick, Mosiuoa Leeto, Benjamin Dewals, William GC Horsnell, Frank Brombacher

**Affiliations:** 1 Division of Immunology, Institute of Infectious disease and Molecular Medicine, Health Sciences Faculty, University of Cape Town, 7925 Cape Town, South Africa

## Abstract

**Background:**

Intestinal mucus production by hyperplasic goblet cells is a striking pathological feature of many parasitic helminth infections and is related to intestinal protection and worm expulsion. Induction of goblet cell hyperplasia is associated with TH2 immune responses, which in helminth infections are controlled primarily by IL-13, and also IL-4. In the study presented here we examine the goblet cell hyperplasic response to three experimental parasitic helminth infections; namely *Nippostrongylus brasiliensis*, *Syphacia obvelata *and *Schistosoma mansoni*.

**Results:**

As expected *N. brasiliensis *infection induced a strong goblet cell hyperplasia dependent on IL-4/IL-13/IL-4Rα expression. In contrast, and despite previously published transiently elevated IL-4/IL-13 levels, *S. obvelata *infections did not increase goblet cell hyperplasia in the colon. Furthermore, induction of goblet cell hyperplasia in response to *S. mansoni *eggs traversing the intestine was equivalent between BALB/c, IL-4/IL-13^-/- ^and IL-4Rα^-/- ^mice.

**Conclusion:**

Together these data demonstrate that intestinal goblet cell hyperplasia can be independent of TH2 immune responses associated with parasitic helminth infections.

## Background

Interleukin (IL)-4 and IL-13 are related cytokines and the dominant mediators of TH2 immune responses [1-3]. Signalling by both cytokines is dependent on binding to heterodimeric receptors containing the IL-4 receptor α chain (IL-4Rα). Ligand binding results in intracellular signalling pathways activating the TH2 defining transcription factors STAT-6 and/or GATA-3 [4,5]. This polarisation to a TH2 immune response is essential for the successful resolution of a number of helminth infections [6-10].

Actual worm expulsion, in nematode infections, is associated with increased IL-13/IL-4Rα/STAT-6 dependent intestinal smooth muscle contractions, epithelial cell turnover and goblet cell hyperplasia [11-13]. Infections of IL-4^-/-^, IL-13^-/-^, IL-4Rα^-/- ^and Stat 6^-/- ^mice with the nematodes *Trichuris muris, Heligmosomoides polygyrus *and *Nippostrongylus brasiliensis *have demonstrated a positive relationship between polarisation to a TH2 immune response, goblet cell hyperplasia and worm expulsion [14-19]. In support of a role for goblet cell derived mucus in worm expulsion *in vitro *experiments have demonstrated increased viscosity of mileu surrounding *N. brasiliensis *at an equivalent density to intestinal mucus inhibits worm movement [20]. Moreover, isolation of the goblet cell secreted protein RELMβ/FIZZ2 and incubation with parasitic nematodes *in vitro *results in impaired chemotactic function in the worm [21]. These observations have led to TH2 induced goblet cell hyperplasia being considered a key mechanistic factor in resolving gastrointestinal related nematode infections [22-24]

Intestinal goblet cell hyperplasia in *Schistosoma mansoni *(*S. mansoni*) infections is driven by parasite eggs traversing the intestine [8,25], as opposed to nematode infections where adult worms residing in the intestine induce the goblet cell responses [8,9,26]. *S. mansoni *eggs produced by adults residing in the mesenteric venules move from the blood vessels through the intestine passing to the lumen. This movement of eggs generates considerable tissue damage as well as inducing a strong mucosal response in the intestine [8,27]. As with nematode infections, *S. mansoni *induced mucus production has been considered to be TH2 dependant [22,28-30].

In this study we examined goblet cell hyperplasia in response to infection with the nematodes *N. brasiliensis *and *Syphacia obvelata *and the trematode *S. mansoni*. As already published *N. brasiliensis *infection induced a goblet cell hyperplasic response dependent on IL-4/IL-13/IL-4Rα expression [9]. However, infection with the nematode *S. obvelata *did not increase goblet cell hyperplasia in the host colon, irrespective of IL-4Rα expression. Such data demonstrates that IL-4Rα driven goblet cell hyperplasia may not be essential for the clearance of all gastro-intestinal nematode infections. Furthermore, we also show *S. mansoni *induced goblet cell hyperplasia to be independent of IL-4/IL-13 responsiveness. This data represents the first demonstration of goblet cell hyperplasia and mucus production in response to helminth infections being independent of IL-4/IL-13.

## Results

### *N. brasiliensis *infection induces IL-4/IL-13 dependent goblet cell hyperplasia while *S. obvelata *infection does not induce goblet cell hyperplasia

Examination of IL-4/IL-13 dependent goblet cell hyperplasic responses in the intestinal niches utilised by the nematodes *N. brasiliensis *and *S. obvelata *infections was carried out in BALB/c, IL-4/IL-13^-/-^, IL-4^-/- ^and IL-4Rα^-/- ^mice.

**Figure 1:**
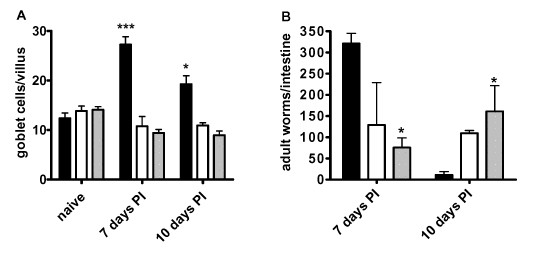
**Goblet cell hyperplasia in *N. brasiliensis *infection**. A) Goblet cell quantification naïve mice and *N. brasiliensis *infected BALB/c (solid bar), IL-4Rα^-/- ^(open bar) and IL-4/IL-13^-/- ^(gray bar) mice 7 days and 10 days PI in small intestine sections. B) Intestinal adult worm burden 7 and 10 days post infection. Data representative of two experiments are shown. Data are means of four mice per group ± SEM. * *P *< 0.05; *** *P *< 0.001 (significantly different from naïve mice).

*N. brasiliensis *infected BALB/c mice demonstrated significantly higher levels of goblet cell hyperplasia in the small intestine at both days 7 and 10 post infection (PI) when compared to naïve mice (Figure 1A). However, no significant increase in the number of goblet cells in the intestine could be detected in infected and naïve IL-4/IL-13^-/- ^or IL4Rα^-/- ^mice when compared to naïve controls (Figure 1A). Examination of intestinal worm burdens in BALB/c mice showed resolution of infection by day 10 PI. Both IL-4/IL-13^-/- ^and IL-4Rα^-/- ^mice failed to expel adult worms by day 10 PI (Figure 1B). These data confirm *N. brasiliensis *clearance to be associated with an IL-4/13/IL-4Rα dependent goblet cell hyperplasia.

**Figure 2:**
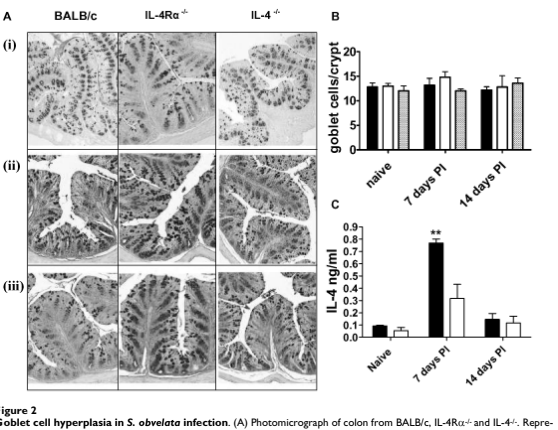
**Goblet cell hyperplasia in *S. obvelata *infection**. (A) Photomicrograph of colon from BALB/c, IL-4Rα^-/- ^and IL-4^-/-^. Representative pictures of colon sections are shown from both naïve (i) and pinworm infected mice at 7 (ii) and 14 days PI (iii). Sections were stained with PAS to identify goblet cells. 100× magnification. (B) Quantification of goblet cells per crypt in colon 7 and 14 days PI in BALB/c (solid bar), IL-4Rα^-/- ^(open bar) and IL-4/IL-13^-/- ^(gray bar). Data representative of two experiments are shown. Data are means of four mice per group ± SEM. (C) *S. obvelata *dependent IL-4 secretion from anti-CD3 restimulated splenocytes. BALB/c (solid bar), IL-4Rα^-/- ^(open bar). **, P < 0.01 (significantly different from naive mice). Data representative of two experiments showing means for four mice/group ± SD.

*S. obvelata *adult worm burdens in infected wild type mice are only detectable by day 28 PI (approx. 1.25 worms/caecum), this burden peaks by day 35 PI to approximately 21 worms/caecum. Infection of IL-4Rα^-/- ^mice results in considerably higher worm burdens (approx. 251 worms/caecum at day 28 PI and 400 worms/caecum at day 35 PI) when compared to wild type mice [10]. In contrast to *N. brasiliensis *infected mice, no induction of goblet cell hyperplasia in the colon of *S. obvelata *infected BALB/c, IL-4Rα^-/- ^and IL-4^-/- ^mice was seen (Figure 2A and 2B). This lack of a goblet cell response was irrespective of heightened levels of the TH2 cytokine IL-4 at day 7 PI (p < 0.01) in BALB/c restimulated splenocytes isolated from infected mice (Figure 2C) BALB/c IL-4 levels declined to that found in naïve mice at day 14 PI. IL-4Rα^-/- ^mice failed to demonstrate any significant increase in IL-4 production when compared to naïve mice.

Together these data demonstrate that *S. obvelata *infections do not induce a colonic mucus response even though levels of IL-4 and other TH2 cytokines are significantly increased [10]

### *Schistosoma mansoni *induces goblet cell hyperplasia in the intestine in an IL-4/IL-13 independent manner

**Figure 3:**
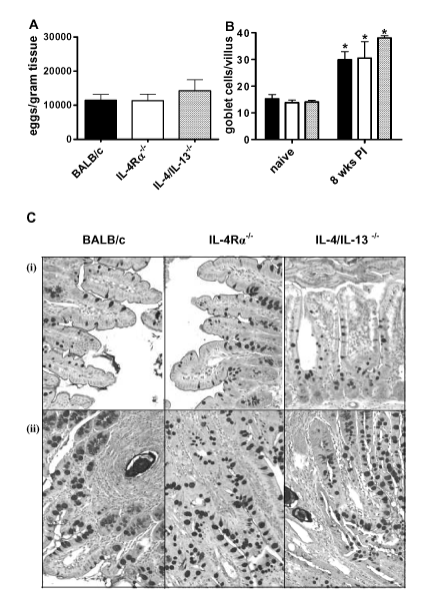
**Goblet cell hyperplasia in ileum during *S. mansoni *infection**. (A) *S. mansoni *egg content in the ileum of BALB/c (solid bar), IL-4Rα^-/- ^(open bar) and IL-4/IL-13^-/- ^(gray bar) mice at 8 weeks PI. Data are pooled from 2 to 4 individual experiments. Data are means of these experiments ± SEM. (B) Quantification of goblet cells per villus in ileum of naïve mice and 8 weeks PI from BALB/c (black bars), IL-4Rα^-/- ^(open bar) and IL-4/IL-13^-/- ^(gray bar). Data representative of three experiments are shown. * *P *< 0.05 (significantly different from naïve mice). (C) Photomicrograph of ileum from BALB/c, IL-4Rα^-/- ^and IL-4/IL-13^-/-^. Representative pictures of ileum sections are shown from both naïve (i) and *S. mansoni *infected mice at 8 weeks PI (ii). Sections were stained with PAS to identify goblet cells. 100× magnification.

**Figure 4:**
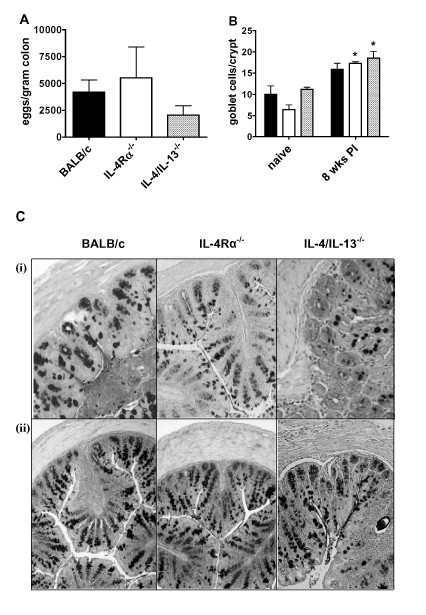
**Goblet cell hyperplasia in the colon during *S. mansoni *infection**. (A) Tissue egg content of the colon from *S. mansoni *infected BALB/c (solid bar), IL-4Rα^-/- ^(open bar) and IL-4/IL-13^-/- ^(gray bar) mice at 8 weeks PI. Data are pooled from 2 to 4 individual experiments. Data are means of these experiments ± SEM. (B) Quantification of goblet cell number per villus in colon of naïve mice and 8 weeks PI from BALB/c (solid bar), IL-4Rα^-/- ^(open bar) and IL-4/IL-13^-/- ^(gray bar) mice. Data representative of three experiments are shown. * *P *< 0.05 (significantly different from naïve mice). (C) Photomicrograph of colon from BALB/c, IL-4Rα^-/- ^and IL-4/IL-13^-/-^. Representative pictures of colon sections are shown from both naïve (i) and *S. mansoni *infected mice at 8 weeks PI (ii). Sections were stained with PAS to identify goblet cells. 100× magnification

*S. mansoni *infection induces a strong TH2 immune response and goblet cell hyperplasia related to parasite egg production [25,31] In order to confirm the role of parasite eggs in induction of goblet cell hyperplasia, we analysed the hyperplasic response at 5 weeks PI (before the peak of egg production) and at the peak of parasite egg production; 8 weeks PI. While no difference in the number of goblet cells could be detected in the intestine of naïve or infected BALB/c at 5 weeks PI (data not shown), a strong induction of goblet cell hyperplasia was detected at 8 weeks PI (Figure 3B and 3C). To establish whether this hyperplasic response was dependent on IL-4/IL-13/IL-4Rα responsiveness we examined the intestines of infected IL-4/IL-13^-/- ^and IL-4Rα^-/- ^mice at 8 weeks PI. Here we found no difference in the numbers of eggs accumulating in the small intestine and large intestine between BALB/c, IL-4/IL-13^-/- ^and IL-4Rα^-/- ^mice (Figure 3A and 4A). Goblet cell hyperplasia in the small intestine of all infected mouse groups was significantly elevated above naïve controls (Figures 3B, C(i) and 3C(ii)). Furthermore, equivalent levels of goblet cell hyperplasia were found in the intestine of all infected mice groups (Figures 3B and 3C). To demonstrate if IL-4/IL-13/IL-4Rα independent goblet cell hyperplasia occurred throughout the intestine we also examined the colon of both naïve and infected mice. As with the small intestine goblet cell hyperplasia was elevated above naïve controls in all mouse groups and no differences were found between infected groups (Figure 4B and 4C). Together these results demonstrate IL-4/IL-13/IL-4Rα independent goblet cell hyperplasia in the intestine of mice infected with *S. mansoni*.

## Discussion

Our data demonstrates that (i) goblet cell hyperplasia is dependent on helminth species and (ii) IL-4/IL-13 responsiveness is not required for induction of *S. mansoni *egg induced goblet cell hyperplasia.

It has previously been demonstrated that *N. brasiliensis *[9] and *S. obvelata *[10] infected IL-4^-/-^, IL-13^-/- ^and IL-4Rα^-/- ^mice have impaired worm expulsion, while in *S. mansoni *infections IL-4/IL-13 signalling is essential for host survival [8]. A common feature of both *N. brasiliensis *and *S. mansoni *infections is the hosts' goblet cell hyperplasic response to the parasite. Such responses have previously been considered to be dependent, in part at least, on the hosts TH2 polarised immune response [9,32]. From the data presented here and in other studies this does indeed appear to be the case in *N. brasiliensis *infections[6,8,9]. Work on other parasitic nematode models such a *T. muris *also show a TH2 dependent worm expulsion and goblet cell response [16]. However, in this study we have demonstrated that this may not be the case for all intestinal nematode infections.

Following oral infection with *S. obvelata *eggs, larvae emerge in the hosts small intestine at 7 day PI [33]. From here the larvae migrate, mature and establish the definitive infection in the hosts cecum and colon. We found the hosts TH2 immune response to peak at day 7 PI and then decreases from at least day 14 PI. Previous work has shown that by day 35 PI this response is undetectable [10]. Together, these data demonstrate a transient TH2 response to this infection. TH2 responses in other intestinal nematode infections result in strong goblet cell hyperplasic responses [14,17-19]. However, mice infected with *S. obvelata *failed to generate hyperplasic goblet cell responses, suggesting that TH2 induction of intestinal mucus responses is not a common feature of intestinal nematode infections, or that the TH2 response needs to be sustained. Other factors such as prostaglandins [34], cholinergic [13] and non-cholinergic [13] agonist may also play a role. Additionally, the different niches occupied by various species of parasitic nematodes could effect the host response to them [35]. *S. obvelata *infections do not cause major pathology in the intestine [36] as opposed to *N. brasiliensis *and *T. muris *which cause considerable histological damage to the hosts intestinal architecture [9,22]. Such differences in worm pathogenicity may explain the lack of a goblet cell response in *S. obvelata *infections, irrespective of the hosts TH2 polarisation [10].

*S. mansoni *infection induces a strong TH2 response initiated by worm egg production at week 4 PI and persists throughout the infection [37]. Associated with this are significant levels of goblet cell hyperplasia in the intestine [25,31]. *S. mansoni *egg antigens have previously been shown to also induce goblet cell hyperplasia in the lung in a IL-4Rα dependent manner [32]. However the role of IL-4Rα in goblet cell hyperplasia in the intestine during the live infection has not been shown. An explanation for the IL-4Rα independent hyperplasia described here could be the mode of *S. mansoni *infection and its interaction with the hosts' tissue. *S. mansoni *eggs cause pathology from the adventitial surface of the intestine, as opposed to nematodes driving the pathology from the lumen. We propose that the severe tissue damage resulting from the eggs migration from the adventitial surface to the lumen is capable of initiating a goblet cell response, independently of IL-4 and IL-13 signalling during *S. mansoni *infection.

In addition to IL-4/IL-13 other cytokines may act to induce goblet cells hyperplasia. IL-9 and IL-5 have previously been shown to play a role in directly inducing IL-4/IL-13 independent goblet cell hyperplasia in lung models [38,39]. IL-9 overexpressing transgenic mice infected with *S. mansoni *do have increased goblet cell hyperplasia [40]. However IL-9 transgenic mice also had increased IL-4 and IL-13 compared to wild type mice, and therefore it cannot be concluded that IL-9 directly increases goblet cell hyperplasia. Furthermore IL-9 levels are decreased in *N. brasiliensis *infected IL-4Rα^-/- ^mice [9]. As such IL-4/IL-13 independent intestinal goblet cell hyperplasia may not be due to increased IL-9. No clear reports linking IL-5 to goblet cell hyperplasia during *S. mansoni *infection have reported. As IL-4Rα^-/- ^mice have decreased IL-5 expression it is also unlikely that IL-5 induces intestinal goblet cell hyperplasia in *S. mansoni *infections [8].

## Conclusion

Our results demonstrate for the first time that intestinal goblet cell hyperplasia in response to parasitic helminth infections can occur independently of IL-4/IL-13 signalling and that intestinal nematode infections may not always induce a goblet cell response.

## Methods

### Mouse strains

IL-4^-/- ^[41] IL-4/13^-/-^[2] and IL-4Rα^-/- ^[3] mice were generated on a BALB/c background. BALB/c mice were used as controls in all experiments. All mice were age and sex matched. Mice were kept in the Health Science Faculty animal unit of the University of Cape Town (UCT), in individually ventilated cages under specific-pathogen-free (SPF) conditions. All experiments were performed in accordance with guidelines laid down by the Animal Ethics Research Board of UCT (Cape Town, South Africa).

### Parasites and infection

#### *Syphacia obvelata*

Infection and recovery of *S. obvelata *were performed as previously described [33]. Briefly, eggs of *S. obvelata *used for infection were collected from the caeca of naturally infected mice (IL-4/13^-/-^, and IL-4Rα^-/-^) maintained in barrier facilities. The caeca were collected in 0.65% NaCl, cut open, and submerged in a gauze mesh at the mouth of a conical flask for 1 to 2 h at 37°C to allow the worms to migrate out. Worm burdens were assessed on various days post infection. After being washed in 0.65% NaCl, worms were crushed and their eggs were isolated by passage through 70 μm nylon cell strainers (BD Falcon, BD Biosciences, Belgium). Each mouse was inoculated orally with 500 eggs using oral dosing cannulae (VetTech, Cheshire, United Kingdom).

#### *Nippostrongylus brasiliensis*

*N. brasiliensis *nematodes were kindly provided by Klaus Erb, (Wurzberg, Germany). Mice were subcutaneously injected with 750 L3 larvae of *N. brasiliensis*. Analysis of numbers of adult worm numbers in the intestine was determined as previously described [9].

#### *Schistosoma mansoni*

Naïve sex-matched mice from 6 to 10 weeks of age were percutaneously infected with 70 to 80 live cercariae of a Puerto Rican strain of *S. mansoni *obtained from infected *Biomphalaria glabrata *snails. Eight weeks post infection the intestine was surgically removed. Ileum and colon were removed 2 cm proximal and 0.5 cm distal to the caecum, respectively [42]. Approximately 2 cm of tissue was weighed and digested in 5 ml of 5% potassium hydroxide overnight at 37°C. The digests were vortexed and centrifuged at 100 g for 5 min to pellet eggs. The supernatant was aspirated until 1–2 mls remained. The eggs were vortexed and counted in 50 μl in triplicate. The counts were presented as eggs per gram of tissue as previously described [43,44]

### Histology

Tissue samples were fixed in a neutral buffered formalin solution. Following embedding in paraffin, samples were cut into 5–7 μm sections. Sections were stained with periodic acid Schiff reagent (PAS). The number of positively stained cells per five villi or crypts was counted by light microscopy for small intestine or colon, respectively. All samples were randomized and counted in a blinded manner. Photomicrographs were captured using a Nikon 5.0 Mega Pixels Color Digital Camera (Digital SIGHT DS-SMc).

### Splenocyte restimulation and IL-4 cytokine ELISA

Single cell splenocyte suspensions were prepared from spleens removed from infected (days 7 and 14 PI) and uninfected mice. 1 × 10^6 ^splenocytes per ml were cultured in IMDM (Gibco) media supplemented with 10% fetal calf serum (Gibco) for 72 h at 37°C in 96 well plates pre-coated with either PBS or 20 mg/ml anti-CD3 (clone 145-2C11). Cells were then centrifuged at 1200 rpm for 5 min and the supernatants collected. Supernatent IL-4 concentrations were then determined by ELISA as described previously [3].

### Statistics

Data are presented as means ± standard error of the mean (SEM), and the significant differences were determined using Student's *t *test (Prism software [45]).

## Abbreviations

The following abbreviations were used: IL, interleukin; KO, knockout; PAS, periodic acid Schiff; PI, post infection; and WT, wild type.

## Authors' contributions

RGM designed the study, carried out the *S. mansoni *infections, histological and statistical analysis of all infection studies and drafted the manuscript. CM carried out the *S. obelata *infections and the associated histological analysis. EMS carried out *N. brasiliensis *infections and the associated histological analysis. LCEF conducted histological stains and contributed to the analysis of all infection studies. ML participated in design of the study and assisted with infections. BD participated in carried out *S. mansoni *infections and drafting of the manuscript. WGCH participated in *N. brasiliensis *infections, participated in interpretation of the data and with drafting of the manuscript, FB conceived of the study, participated in its design, coordination, interpretation of the data and drafting the manuscript. All authors read and approved the final manuscript.
